# Complete Genome Sequence of Austwickia chelonae LK16-18, Isolated from Crocodile Lizards

**DOI:** 10.1128/MRA.01140-18

**Published:** 2018-10-25

**Authors:** Hai-Ying Jiang, Ming-Wei Huang, Li-Bo Lin, Nan He, Jin-Ping Chen

**Affiliations:** a Guangdong Key Laboratory of Animal Conservation and Resource Utilization, Guangdong Public Laboratory of Wild Animal Conservation and Utilization, Guangdong Institute of Applied Biological Resources, Guangzhou, China; b Guangdong Luokeng *Shinisaurus crocodilurus* National Nature Reserve, Shaoguan, China; University of California, Riverside

## Abstract

Austwickia chelonae, a species of Actinobacteria, is one of the pathogens that cause dermatophilosis in animals. Here, we report the complete genome sequence of Austwickia chelonae LK16-18, which was isolated from cutaneous granulomas in crocodile lizards.

## ANNOUNCEMENT

Austwickia chelonae belongs to the family *Dermatophilaceae* (phylum *Actinobacteria*). It is one of the pathogens that causes dermatophilosis (1, 2). It has a wide range of hosts, such as snapping turtles, king cobras, and bearded dragons (3–6). In this study, A. chelonae was isolated from a cutaneous granuloma in the crocodile lizard (Shinisaurus crocodilurus), a critically endangered reptile. This disease has caused many deaths in crocodile lizards. The genome sequence generated for A. chelonae was a useful data point in the understanding of the pathogenic mechanism of this actinobacterium.

Austwickia chelonae LK16-18 was isolated from a cutaneous granuloma in a crocodile lizard. The strain was cultured on Columbia blood agar base plates at 30°C for 48 h and then transferred into Columbia medium fermented for 48 h in order to obtain more bacterial cells. The whole genome of A. chelonae LK16-18 was extracted using a TIANamp bacteria DNA kit (catalog number DP302; Tiangen Biotech Co., Ltd., Beijing, China) with 20 mg/ml lysozyme (Tiangen Biotech Co., Ltd.). DNAs were interrupted into 10-kb fragments by using a Covaris g-TUBE. A SMRTbell library was constructed using a SMRTbell template prep kit 1.0 (Pacific Biosciences, USA). Sequencing was performed using a PacBio system at Beijing Novogene Bioinformatics Technology Co., Ltd. The low-quality reads were filtered, and the clean data were assembled to generate one contig without gaps using SMRT Link v5.0.1 software. Reads were mapped onto the assembled genome sequence to calculate the distribution of sequencing depths. In addition, Illumina 150-bp paired-end sequencing was performed in order to correct the PacBio sequencing results. The library for Illumina sequencing was prepared using a TruSeq DNA PCR-free sample preparation kit (Illumina, Inc., USA). For genome annotation, GeneMarkS v4.28 (7) was used to retrieve the related coding genes, RepeatMasker v4.0.7 (http://www.repeatmasker.org/) was used to predict interspersed repetitive sequences, Tandem Repeats Finder (TRF) v4.09 was used to analyze tandem repeats, tRNAscan-SE v2.0 (8) was used to predict tRNA genes, RNAmmer v1.2 (9) was used to analyze rRNA genes, a BLAST search against the Rfam database (10) was performed to predict small nuclear RNAs (snRNAs), IslandPath-DIMOB v1.0.0 (11) was used to predict genomic islands (GIs), TransposonPSI was used to predict the transposons, PHAST (12) was used for prophage prediction (http://phast.wishartlab.com/), and CRISPRFinder was used for clustered regularly interspaced short palindromic repeat (CRISPR) sequence identification. For gene function annotation, the DIAMOND v0.9.16 program (13), with an E value of ≤1 × 10^−5^, was used to align against seven databases, namely, the Gene Ontology (GO), Kyoto Encyclopedia of Genes and Genomes (KEGG), Clusters of Orthologous Groups (COG), nonredundant protein (nr) databases, Pfam, the Transporter Classification Database (TCDB), and Swiss-Prot, and the item with the highest score was selected. In addition, the pathogenicity and drug resistance analyses were performed using the Pathogen Host Interactions (PHI) database, the Virulence Factors of Pathogenic Bacteria Database (VFDB), and the Antibiotic Resistance Genes Database (ARDB).

The total number of PacBio reads was 195,458, with an *N*_50_ value of 8,532 bp. The genome of A. chelonae LK16-18 was assembled into a circular chromosome (3,628,119 bp) and a plasmid (3,851 bp), and the G+C genome contents were 64.39% and 60.4%, respectively. A total of 1,337,730,976 bp of clean data were generated. The genome coverage was ∼368×. The genome of A. chelonae LK16-18 contained 3,259 coding genes and 6 rRNA genes. In addition, 4 CRISPR sequences, 11 GIs, 1 prophage sequence, 29 interspersed repeat sequences, and 393 tandem repeat sequences were found. For noncoding RNAs, two 5S rRNAs, two 16S rRNAs, and two 23S rRNAs were found, whereas tRNA was not found.

### Data availability.

The whole-genome sequences of A. chelonae LK16-18 have been deposited in the GenBank database under SRA number SRP162086 and assembly numbers CP031447 and CP031448. The versions described in this paper are the first versions. The BioProject number for this study is PRJNA484703.
